# Salmagundi

**Published:** 1888-03

**Authors:** 


### SALMAGUNDI.

EDITED BY E. G. BETTY, D.D.S.

PROF. RAMSAY is of the opinion that color-blindness is a fault of the brain and not of the eye.

A MONUMENT is to be erected in Köping, Sweden, to the memory of Scheele, the discoverer of oxygen.

IN THE PURE FOOD CONVENTION at Washington, on January 20, resolutions were adopted urging the immediate enactment of laws to prevent the adulteration of foods, drinks and drugs. The convention refused to recommend special legislation in regard to lard and oleomargarine. A permanent National Pure Food Association was organized and officers wers elected for the ensuing year. A vice-president from each State represented in the convention was also elected. The old Committee on Legislation was continued, but was strengthened by the addition of the Present, three secretaries and the treasurer.

Antiseptic Mouth-Wash.--One of the greatest living authorities upon buccal bacteriology, Dr. Miller, finds that by using the following mixture, he could completely steralize the mouth, cavities in carious teeth, etc.:

| | |
|---|---|
| Thymol | 4 grains. |
| Benzoic acid | 45 " |
| Tincture of eucalyptus | 3½fld. drachms. |
| Water | 25 fld. os. |

The mouth to be will rinsed with this mixture, especially before going to bed.

For retail, a mixture of water and spirit is required for a presentable preparation, and it should be made much stronger, say 5, instead of 25 oz., and diluted when required.—*Chem. and Druggist.*

---

Younger's Operation of Implantation revives some curious matters of interest connected with the old practice of trans-transplantation, much in vogue near a hundred years ago, both in this country and England, and advocated by Hunter. It appears, according to L. S. Parmly, in his work on "The Natural History and Management of the Teeth," 1820, that "the operation is extremely limited, being confined to only the front teeth, or those having single fangs." However this may be, there was in England, about the beginning of this century, a great demand for such teeth, and they could not always be procured from the living subject. The consequence was that the dead were surreptitiously despoiled of their teeth to serve the unfortunate living; witness the following from the "Book of Days," an extract from an article relating to the "body-snatching" proclivities of Sir Astley Cooper, the champion of dentist Parmly in London. "The graves were not always disturbed to obtain possession of the entire body, for the *teeth* (italics not ours) alone, at one time offered tempting remuneration. Mr. Cooper (Sir Astley), relates an instance of a ressurrectionist, feigning to look out a burial place for his poor wife, and thus obtaining access to the vault of a meeting house, the trap-door of which he unbolted, so that at night he let himself down into the vault, and secured the front teeth of the whole congregation, by which he cleared £60."

New Blistering Liquid.—D. Boni (*Giorn farm. trent.*) recommends chloral camphor as superior to any of the ordinary vehicles for the active principle of cantharides. His preparation consists of

| | |
|---|---|
| Camphor | 20 parts. |
| Chloral hydrate | 30 " |
| Cantharides | 10 " |

The pulverized camphor is mixed with the chloral hydrate and heated to 140° F. until fused, the bruised catharides is then added, and the mixture digested at 140° to 158° F. one hour, with occasional stirring, then strained and preserved in a glass stoppered bottle. It is to be applied by a compress, or in the case of children or delicate women, simply penciled over the surface. The circumstance that it is non-volatile gives it a great advantage over cantharidal collodion.—*Quarterly Therap. Review*, January 1, 1888.

The Teeth in Congenital Syphilis.—At a late meeting of the London Harveian Society, Dr. J. Hutchinson, Jr., spoke on this topic. The fallacy of looking for characteristic signs in the temporary teeth was dwelt upon, their liability to premature necrosis and falling out being illustrated by specimens. Those teeth which in after-life showed syphilitic deformity in the greatest degree were those which calcified first, and which during the year lay nearest to the gum surface, namely, the permanent incisors and first molars. The three small projections at the cutting edge of the central incisors seen in normal teeth were noticed, and the wearing away or non-development of the median one was described in connection with the formation of the semi-lunar notch and the narrowed cutting edge. The horizontal erosion of the enamel, resulting from mercurial stomatitis in infancy, and its occasional coincidence with true syphilitic malformation of the teeth were illustrated, and stress laid on the fact that the upper central incisors were the real test teeth of inherited syphilis, though many children with the inherited disease presented perfectly normal teeth. No explanation could at present be given for the preponderance of female over male patients observed in connection with inherited syphilitic disease of the eyes and auditory system. Dr. Hughlings Jackson believed that a wide and exact knowledge in the profession of the particular and decisive signs of inherited syphilis mentioned would, by leading to early and definite treatment, save many people from terrible calamities.—*The Medical Record.*

